# Whole Exome Sequencing Identifies Genes Associated With Non-Obstructive Azoospermia

**DOI:** 10.3389/fgene.2022.872179

**Published:** 2022-04-13

**Authors:** Hongguo Zhang, Wei Li, Yuting Jiang, Jia Li, Mucheng Chen, Ruixue Wang, Jing Zhao, Zhiyu Peng, Hui Huang, Ruizhi Liu

**Affiliations:** ^1^ Reproductive Medicine and Prenatal Diagnosis Center, The First Hospital, Jilin University, Changchun, China; ^2^ BGI Genomics, BGI-Shenzhen, Shenzhen, China

**Keywords:** causative genes, linkage analysis, non-obstructive azoospermia, whole exome sequencing, rare variant association study

## Abstract

**Background:** Non-obstructive azoospermia (NOA) affects nearly 1% of men; however, the landscape of the causative genes is largely unknown.

**Objective:** To explore the genetic etiology which is the fundamental cause of NOA, a prospective case-control study and parental–proband trio linkage analysis were performed.

**Materials:** A total of 133 patients with clinicopathological NOA and 343 fertile controls were recruited from a single large academic fertility center located in Northeast China; in addition, eleven trio families were available and enrolled.

**Results:** Whole exome sequencing-based rare variant association study between the cases and controls was performed using the gene burden association testing. Linkage analysis on the trio families was also interrogated. In total, 648 genes were identified to be associated with NOA (three of which were previously reported), out of which six novel genes were found further associated based on the linkage analysis in the trio families, and involved in the meiosis-related network.

**Discussion and Conclusion**: The six currently identified genes potentially account for a fraction (3.76%, 5 out of 133 patients) of the heritability of unidentified NOA, and combining the six novel genes and the three previously reported genes together would potentially account for an overall 6.77% (9 out of 133 patients) heritability of unidentified NOA in this study.

## Introduction

Approximately 7% of the male population worldwide suffer from infertility, and one of the main causes is azoospermia, which is a condition contributing to male infertility with the highest frequency of known genetic factors (about 25%) (Krausz and Riera-Escamilla 2018; Rodrigues, Polisseni et al., 2020). Given that nearly 50% of azoospermia cases are estimated to be associated with genetic defects, the etiology of azoospermia is still very elusive (Rodrigues, Polisseni et al., 2020). In terms of pathobiological mechanism, azoospermia usually is categorized into two groups: non-obstructive azoospermia (NOA) which is defined as no spermatozoa due to failure of spermatogenesis and obstructive azoospermia which occurs due to an obstruction of the seminal ducts and is characterized by the absence of spermatozoa in the ejaculate despite the normal spermatogenesis.

NOA is the most severe form of azoospermia; currently, no treatment can restore spermatogenesis in the majority of patients; however, some patients can benefit from treatment with assisted reproductive technologies. NOA is a heterogenous condition with variable histopathological phenotypes: the Sertoli cell-only syndrome (SCOS) is characterized by a complete absence of germ cells, while spermatogenic arrest is a condition originating from the physiological spermatogenesis defect and is known as the interruption of germinal cell formation of specific cellular type.

Approximately 20% of NOA cases were caused by detectable structural anomalies including chromosomal abnormalities such as the sex-chromosome aneuploidy, the trans-locational aberrations, and microdeletions of azoospermia factors (AZFs) (Krausz and Riera-Escamilla 2018). In addition, monogenic variations have been identified to be a causative for or associated with NOA; the attested genes include X chromosome linked genes TEX11 (Yatsenko, Georgiadis et al., 2015), FAM47C (Chertman, Arora et al., 2019), MAGEB4 (Okutman, Muller et al., 2017), and AR (Mou and Gui 2016); and autosomal genes include PIWIL1 (Gou, Kang et al., 2017), BRCA2 (Zhoucun, Zhang et al., 2006), BOLL and DAZL (Zhang, Xue et al., 2016), TEX15 (Okutman, Muller et al., 2015), SYCP3 (Miyamoto, Hasuike et al., 2003), NR5A1 (Safari, Zare-Abdollahi et al., 2014), SOHLH1 (Choi, Jeon et al., 2010), WT1 (Wang, Li et al., 2013; Xu, Jiang et al., 2017), TAF4B and ZMYND15 (Ayhan, Balkan et al., 2014), FANCM (Kasak, Punab et al., 2018), SPINK2 (Kherraf, Christou-Kent et al., 2017), TEX14 (Gershoni, Hauser et al., 2017), DNAH6 (Gershoni, Hauser et al., 2017), MEIOB (Gershoni, Hauser et al., 2017), RNF212 and STAG3 (Riera-Escamilla, Enguita-Marruedo et al., 2019), XRCC2 (Zhang, Li et al., 2019), TDRD7 and TDRD9 (Arafat, Har-Vardi et al., 2017; Tan, Tu et al., 2019), DMC1 (He, Tu et al., 2018), SYCE1 (Maor-Sagie, Cinnamon et al., 2015), NPAS2 (Ramasamy, Bakircioglu et al., 2015), MCM8 (Tenenbaum-Rakover, Weinberg-Shukron et al., 2015), MEI1 (Ben Khelifa, Ghieh et al., 2018), and STX2 (Nakamura, Kobori et al., 2018). Despite these efforts, the etiology remains unknown in an enormously large part of NOA patients (Krausz and Riera-Escamilla 2018). Considering that a surprisingly large proportion of all genes (roughly 1 in 25 of all mammalian genes) are specifically expressed in the testis, hundreds of genes may be involved in the spermatogenesis process (Schultz, Hamra et al., 2003; Bell, Mello et al., 2020). Therefore, the physiological pathway complexity may contribute to a large number of unresolved patients.

However, current tools for understanding the unidentified genetic etiology of NOA are very limited due to the lack of large pedigrees with infertility; therefore, the discovery of novel genetic factors for unexplained NOA is still the main challenge. In this study we performed whole exome sequencing (WES), one of the most promising and powerful approach for exploring the pathogenic factors of inherent diseases, on 133 unrelated patients and 343 independent controls for rare variant association study (RVAS) (Lee, Abecasis et al., 2014; Artomov, Stratigos et al., 2017; Locke, Steinberg et al., 2019), which was a kind of analysis derived from genome-wide association study (GWAS); meanwhile, 11 trios were analyzed in a maternal inherited or a *de novo* pattern to delineate the causative genes. Protein-protein interaction (PPI) network functional enrichment analysis was also applied to provide potential evidence to further address the abovementioned questions.

## Results

**FIGURE 1:**
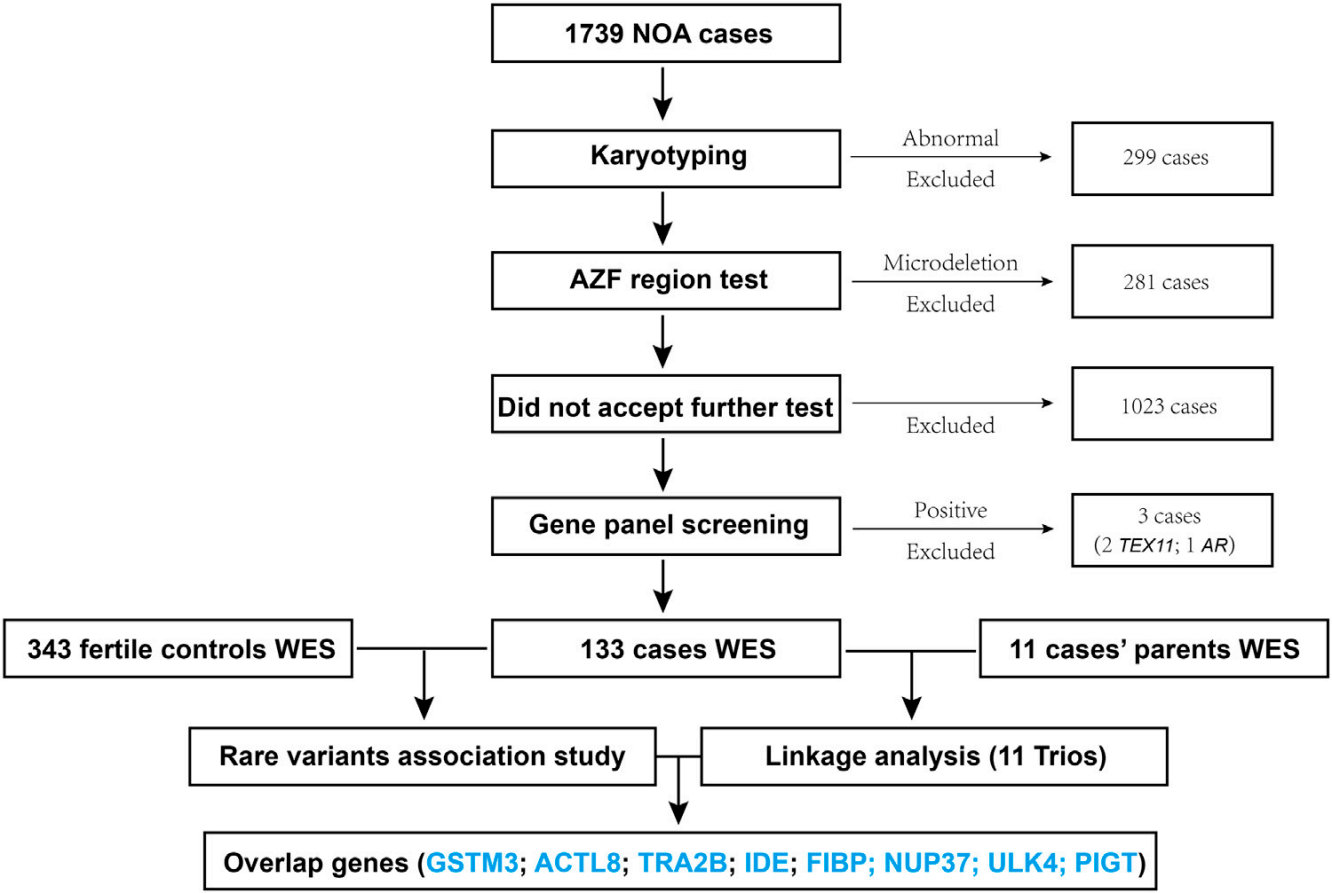
Enrollment of the patients and the workflow diagram for this study.

The workflow diagram of this study is shown in Figure 1, a total of 1739 patients were enrolled, amidst which 299 cases (about 17%) with abnormal karyotyping results (such as sex-autosome translocation, and Y chromosome polymorphisms (Yqh–or Yqh+)) were excluded, followed by 281 cases (about 16%) exclusion for harboring the AZF region (AZFa, AZFb, or AZFc) microdeletions; then 1,023 cases who did not consent to accept further sequencing test were not included. Furthermore, a custom panel for detecting the known genes of azoospermia (Supplementary Table S2) was developed, from which three cases with positive results (two with *TEX11* mutation, one with *AR* mutation) were excluded. Eventually, 133 patients and 343 controls were subjected to WES; linkage analysis for 11 trios was also performed in this study.

Among the 133 patients, WES identified 7 LOFs in six known genes (Table 1). Since *FAM47C* was identified to be highly associated with NOA previously, the X chromosome-linked (XL) *FAM47C* mutation (p.Val118Leufs*40, frameshift mutation similar with previous literature (Chertman, Arora et al., 2019)) from patient 58 in our study was also identified as the potential genetic etiology accordingly; the *BRCA2* mutation in patient 58 (p.Ser 1955*) and patient 82 (p.Thr2197_Glu2198delinsLys) may be associated with NOA. The causative gene *SYCP3* mutation in patient 88 (c.-13-2A > C) may be pathogenic (functional evidence needed) according to an autosomal dominant (AD) inheritance pattern. In autosomal recessive (AR) inheritance, the other genes are uncertainly causative concerning the individuals owing to another pathogenic allele not determined. Pathogenic CNVs were not identified among the 133 patients under WES.

**TABLE 1:** Attested causative genes mutations identified by WES in our study.

Patients	Genes	Accession number	HGVS_c (pLOF)	HGVS_p	Location	Sanger validation	Inheritance	Effect annotation	Zygosity	Chromosome
P19	TEX15	NM_031271.3	c.3098ATGCAATAAT [2 > 1]	p.Cys1037Serfs*47	Exon 1	True	AR	Frameshift	Het	chr8
P58	BRCA2	NM_000059.3	c.5864C > G	p.Ser 1955*	Exon 11	True	AD	Nonsense	Het	chr13
P58	FAM47C	NM_001013736.3	c.351_352insCTTC	p.Val118Leufs*40	Exon 1	True	XL	Frameshift	Hemi	chrX
P80	MEIOB	NM_001163560.2	c.1A > G	p.0?	Exon 2	True	AR	Start codon loss	Het	chr16
P82	BRCA2	NM_000059.3	c.6590_6592delCTG	p.Thr2197_Glu2198delinsLys	Exon 11	True	AD	Indel	Het	chr13
P88	SYCP3	NM_153,694.4	c.-13-2A > C		Intron 1	True	AD	Splice acceptor	Het	chr12
P101	TDRD7	NM_014290.2	c.3121C > T	p.Arg1041*	Exon 17	True	AR	Nonsense	Het	chr9

HGVS, Human Genome Variation Society.

**FIGURE 2:**
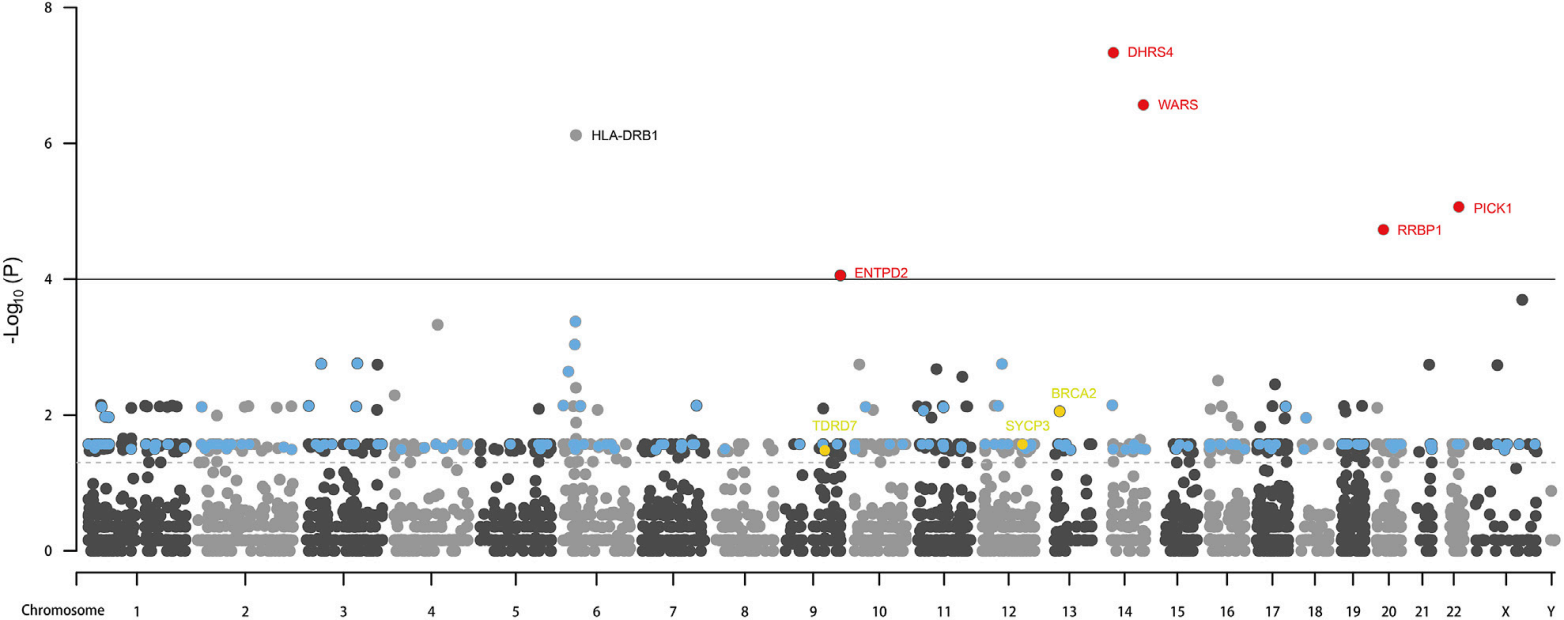
RVAS analysis results. Red, the most significant genes with *p* value lower than 0.0001 identified by RVAS test, and with OR values higher than 1. Blue: the significant genes with *p* value between 0.05 and 0.0001 identified by the RVAS test, with NE higher than 16 (taking the previously reported genes as a reference), and with OR values higher than 6 or with OR values not estimable. Yellow: the significant genes with *p* values between 0.05 and 0.0001 identified by the RVAS test while overlapping with known genes. The solid line denotes the *p* value equals 0.0001; the dashed line denotes the *p* value equals 0.05.

**FIGURE 3:**
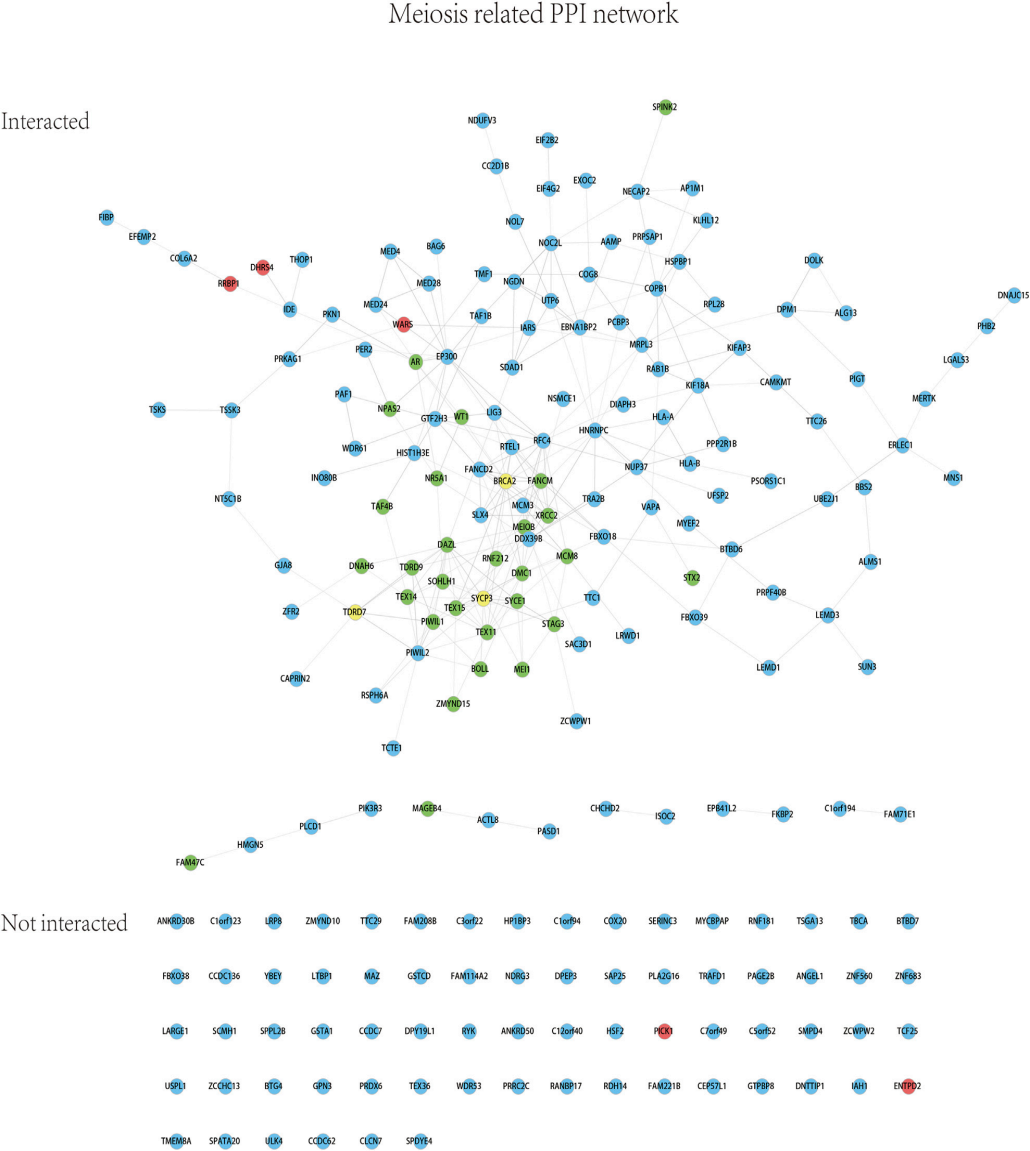
Meiosis-related PPI network based on the RVAS analysis. Red, blue, and yellow: the color code is identical with that in Figure 2. Green: 28 previously known proteins associated with NOA.

Whole exome-wide RVAS results were summarized in Supplementary Table S3, including the *p*-values and the OR values, the normalized expression (NE) of genes in testis tissue from the Human Protein Atlas database were also provided. NE was calculated according to the transcript profiling based on a combination of three transcriptomics datasets HPA, GTEx, and FANTOM5 (genes with no NE data were ruled out in this study). We quantified genes with the OR value >1 or with OR value not estimable due to the absence of the rare variations among the controls, meanwhile we employed a flexible cutoff of *p* < 0.05 due to the statistical power limitation of the relatively small sample size (133 patients) and the low proportion of rare variations among the entire samples (a lot of rare variations absent in controls). As a result, a total of 648 genes (65 genes with *p* < 0.01) were identified to be associated with NOA (three of which were previously reported). The most significant genes reported were *DHRS4*, *WARS1*, *PICK1*, *RRBP1*, and *ENTPD2* (red), whereas the other 179 significant genes were shown in blue; meanwhile, three known genes *BRCA2*, *SYCP3*, and *TDRD7* (yellow) were also identified by the RVAS test in our study (Figure 2).The 187 genes identified by RVAS (red, blue, and yellow) and 28 previously reported proteins (green) were analyzed for the PPI network as shown in Figure 3. Interestingly, the previously reported proteins, except for only MAGEB4 and FAM47C, tended to be involved in the meiosis-related network, which is centered around PIWIL1, TEX11, TEX15, DAZL, and TEX14, etc. The majority of RVAS identified proteins (117), such as WARS, RRBP1, DHRS4 (red), and PIWIL2, DDX39B, SLX4, HNRNPC (blue), were involved and enriched in the meiosis-related PPI network (FDR value for the male gamete generation pathway was 9.56 × 10^–31^), thereby betraying the functional potential to be strongly associated with NOA; whereas 70 proteins, like PICK1 and ENTPD2 (red), were not interacted with the PPI network, attenuating the possibility that these genes play major functional roles in the development of NOA.

**FIGURE 4:**
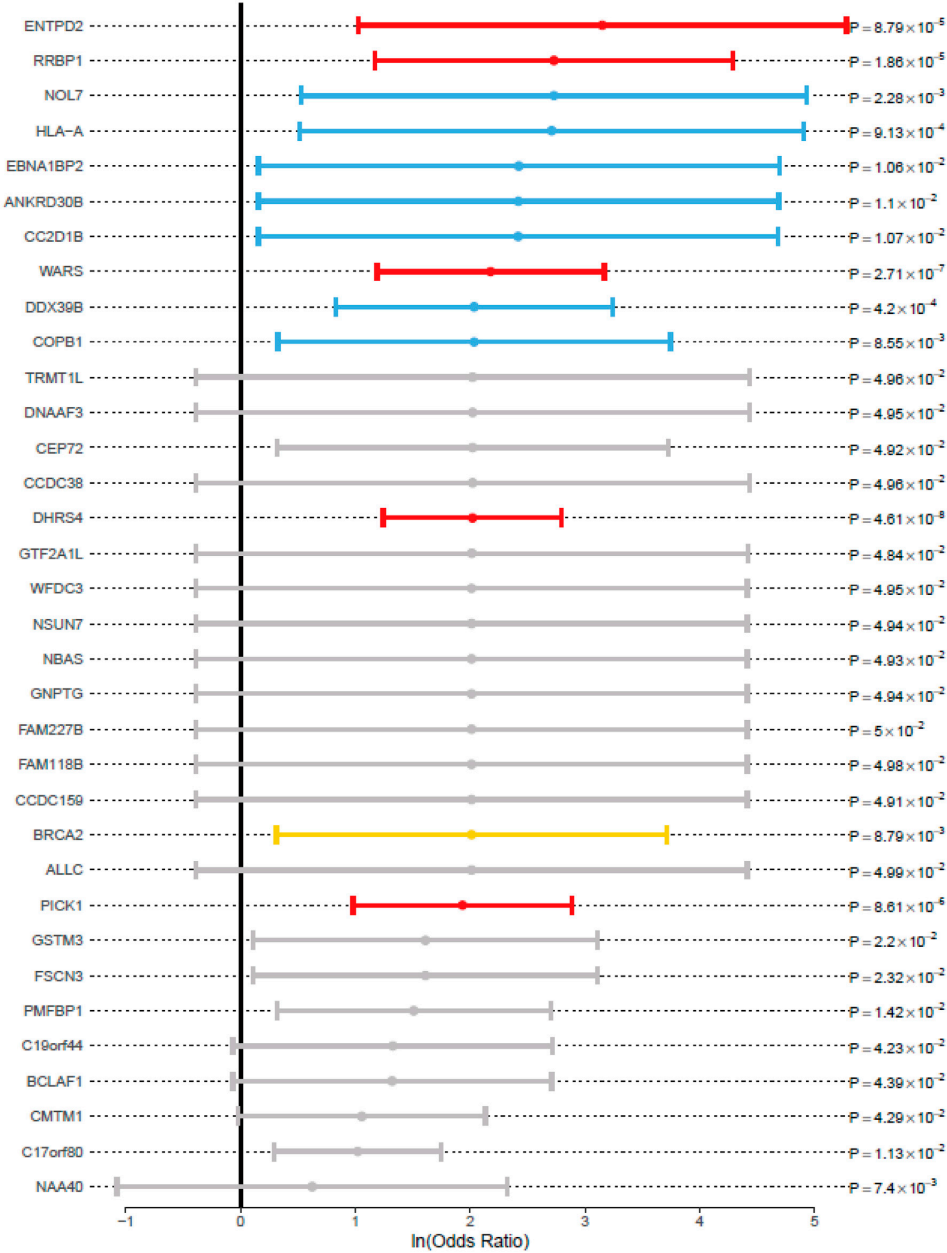
Associated genes with odds ratio and *p* value for the RVAS analysis. 95% CI (whiskers) was shown as the solid line around the ln (odds ratio) value dot (circles). The black vertical line indicates an OR value of 1. The color description was identical with that in Figure 3.

For association study, OR values were not estimable for most of the 187 genes (Supplementary Table S3) due to the absence of rare variations among the controls, we summarized a short list of genes with OR values to evaluate the genetic risk (Figure 4). All the genes with *p* values lower than 0.05, with the testis expression (NE > 16), and with the OR values higher than 1 were included. Consistent with the previous literature, BRCA2 (OR, 7.48; 95% CI, 1.37–40.89; *p* value, 8.79 × 10^–3^) was identified to be associated with NOA in our study. The following genes with high OR values were also involved in the meiosis-related PPI network (Figure 3), including *ENTPD2* (OR, 23.34; 95% CI, 2.78–195.61; *p* value, 8.79 × 10^–5^) which had the highest OR; while *RRBP1* (OR, 15.32; 95% CI, 3.23–72.57; *p* value, 1.86 × 10^–5^), *WARS* (OR, 8.83; 95% CI, 3.29–23.71; *p* value, 2.71 × 10^–7^), *DHRS4* (OR, 7.54; 95% CI, 3.46–16.42; *p* value, 4.61 × 10^–8^), *NOL7* (OR, 15.32; 95% CI, 1.70–138.23; *p* value, 2.28 × 10^–3^), *HLA-A* (OR, 15.03; 95% CI, 1.67–134.84; *p* value, 9.13 × 10^–4^), *EBNA1BP2* (OR, 11.28; 95% CI, 1.17–108.90; *p* value, 1.06 × 10^–2^), *CC2D1B* (OR, 11.22; 95% CI, 1.17–108.12; *p* value, 1.07 × 10^–2^), *DDX39B* (OR, 7.64; 95% CI, 2.28–25.58; *p* value, 4.20 × 10^–4^), and *COPB1* (OR, 7.64; 95% CI, 1.38–42.19; *p* value, 8.55 × 10^–3^) were shown.

In total, 11 trio families were recruited as shown in Figure 1, and their data were analyzed and summarized in Table 2. For linkage analysis, according to a maternally inherited pattern or the *de novo* mutation mechanism to which the gender-specific disease NOA condition was supposed to be genetically attributed, the paternal inherited variations were excluded; meanwhile, the probands’ LOFs were preserved only if the LOFs were not found in the 343 male controls. Sanger sequencing was performed for validating the variations. To facilitate the screening for the mutations, the *p*-value and OR value of RVAS for each gene were also rendered. Eventually, six genes (in bold and double-underlined in Table 2), *ACTL8* and *TRA2B* for patient 62, *IDE* for patient 117, *FIBP* for patient 124, *NUP37* for patient 130, and *PIGT* for patient 131 were regarded as the top candidates of associated genes (*p* < 0.05; OR values not estimable due to the absence of variations among the controls) based on the RVAS test (Figure 2) and their involvement in the meiosis-related PPI network (Figure 3).

**TABLE 2:** Novel gene mutations identified in the trio families.

Proband	Pedigree (trio)	Gene	HGVS_c (pLOF)	HGVS_p	Accession number	Inheritance	Effect annotation	Zygosity	Chromosome	Sanger validation	Normalized expression (testis)	*p* value (RVAS)	Odds ratio (RVAS)	Proteins interaction map
P6	Proband and parents	PRPF40B	c.349-2A > C	NA	NM_001031698.2	De novo	Splice acceptor	Heterozygous	chr12	False	25.1	0.0018	Not estimable	Linked
P38	Proband and parents	LAMA4	c.5336delG	p.Ser1779Thrfs*12	NM_002290.4	Inherited from mother	Frameshift	Heterozygous	chr6	True	9.4	0.2880	3.7321	NA
		NDUFAF1	c.383G [5 > 4]	p.Glu131Lysfs*8	NM_016013.3	Inherited from mother	Frameshift	Heterozygous	chr15	True	14.1	0.0268	Not estimable	NA
		UMODL1	c.3474+1G > C	NA	NM_001199528.2	Inherited from mother	Splice donor	Heterozygous	chr21	True	3.8	0.0855	1.4615	NA
		ZNF442	c.843A [2 > 1]	p.Arg282Aspfs*59	NM_030824.2	Inherited from mother	Frameshift	Heterozygous	chr19	True	3.5	0.0771	2.6689	NA
P48	Proband and parents	EIF4G2	c.1299+2delT	NA	NM_001172705.1	De novo	Splice donor	Heterozygous	chr11	False	60.5	0.0268	Not estimable	Linked
P52	Proband and parents	SEMA3F	c.2161G [2 > 1]	p.Gly721Alafs*10	NM_004186.3	Inherited from mother	Frameshift	Heterozygous	chr3	True	3.1	0.0338	Not estimable	NA
		TMEM63A	c.1578T [6 > 7]	p.Phe529Leufs*6	NM_014698.2	Inherited from mother	Frameshift	Heterozygous	chr1	True	3	0.0075	Not estimable	NA
P60	Proband and parents	ADAM18	c.56-2A > G	NA	NM_014237.2	Inherited from mother	Splice acceptor	Heterozygous	chr8	True	42.7	0.7096	1.3975	NA
		BICC1	c.482A [3 > 2]	p.Thr162Profs*24	NM_001080512.1	Inherited from mother	Frameshift	Heterozygous	chr10	True	4.9	0.0307	Not estimable	NA
		GSTM3	c.48+2T > G	NA	NM_000849.4	Inherited from mother	Splice donor	Heterozygous	chr1	True	96	0.0220	5.0025	NA
		KRT10	c.1458_1459delCC	p.His487Argfs*93	NM_000421.3	De novo	Frameshift	Heterozygous	chr17	NA	9.6	0.3695	1.8646	NA
		SPRR2F	c.218A > G	p.*73*	NM_001014450.1	Inherited from mother	Stop-retained	Heterozygous	chr1	True	2.2	0.0307	Not estimable	NA
		TMEM19	c.738_739insTAGACATTTTTGT	p.Asp247*	NM_018279.3	Inherited from mother	Frameshift	Heterozygous	chr12	True	7	0.2906	3.7321	NA
P62	Proband and parents	ABCB8	c.516_517delTG	p.Glu173Valfs*54	NM_007188.3	Inherited from mother	Frameshift	Heterozygous	chr7	True	13.6	0.0268	Not estimable	NA
		ACTL8	c.761_771delTGGCTCCTGAG	p.Val254Aspfs*4	NM_030812.2	Inherited from mother	Frameshift	Heterozygous	chr1	True	23.6	0.0268	Not estimable	Linked
		ARMC4	c.393C [4 > 5]	p.Ile133Hisfs*8	NM_018076.2	Inherited from mother	Frameshift	Heterozygous	chr10	True	22	0.3036	3.7321	NA
		CDH19	c.2059C > T	p.Gln687*	NM_021153.3	Inherited from mother	Nonsense	Heterozygous	chr18	True	1.9	0.2834	3.7321	NA
		MICA	c.801G > A	p.Trp267*	NM_001177519.1	Inherited from mother	Nonsense	Heterozygous	chr6	True	24.6	0.2916	3.7321	NA
		PRRG4	c.605C > G	p.Ser202*	NM_024081.5	Inherited from mother	Nonsense	Heterozygous	chr11	True	1	0.0268	Not estimable	NA
		RPUSD4	c.651+1G > A	NA	NM_032795.2	Inherited from mother	Splice donor	Heterozygous	chr11	True	7	0.0075	Not estimable	NA
		TRA2B	c.1A > G	p.0?	NM_004593.2	De novo	Start codon loss	Heterozygous	chr3	True	38.4	0.0268	Not estimable	Linked
		ZNF221	c.1309C > T	p.Arg437*	NM_013359.2	Inherited from mother	Nonsense	Heterozygous	chr19	True	3.5	0.0268	Not estimable	NA
P98	Proband and parents	C1orf87	c.887C > G	p.Ser296*	NM_152,377.2	Inherited from mother	Nonsense	Heterozygous	chr1	True	1.2	1.0000	1.2411	NA
		GNPTAB	c.98C [2 > 1]	p.Ala34Profs*49	NM_024312.4	Inherited from mother	Frameshift	Heterozygous	chr12	True	7.4	0.0342	5.6087	NA
		MAGEC1	c.721dupT	p.Ser241Phefs*14	NM_005462.4	De novo	Frameshift	Hemizygous	chrX	NA	23.5	0.7476	0.6761	NA
		OR5C1	c.527G [2 > 1]	p.Lys177Argfs*40	NM_001001923.1	Inherited from mother	Frameshift	Heterozygous	chr9	True	1	0.0512	7.4924	NA
		RNF224	c.1A > G	p.0?	NM_001190228.1	Inherited from mother	Start codon loss	Heterozygous	chr9	True	1.2	0.3003	3.7321	NA
		SELP	c.2394T [3 > 1]	p.Phe799Glnfs*5	NM_003005.3	Inherited from mother	Frameshift	Heterozygous	chr1	True	3.1	0.0268	Not estimable	NA
P117	Proband and parents	CD109	c.3911+1G > T	NA	NM_133,493.3	Inherited from mother	Splice donor	Heterozygous	chr6	True	3.3	0.1300	3.7321	NA
		DDX11	c.577G [4 > 2]	p.Glu194Glyfs*11	NM_030653.3	Inherited from mother	Frameshift	Heterozygous	chr12	True	8.2	0.0072	Not estimable	NA
		IDE	c.1484G > A	p.Trp495*	NM_004969.3	Inherited from mother	Nonsense	Heterozygous	chr10	True	18.1	0.0268	Not estimable	Linked
		NALCN	c.4905+1G > C	NA	NM_052867.2	Inherited from mother	Splice donor	Heterozygous	chr13	True	2	0.2895	3.7321	NA
		PLAC9	c.28G > T	p.Gly10*	NM_001012973.1	De novo	Nonsense	Heterozygous	chr10	False	8.4	0.0268	Not estimable	NA
		POLD2	c.885+2T > C	NA	NM_006230.3	Inherited from mother	Splice donor	Heterozygous	chr7	True	14.4	0.0268	Not estimable	NA
		VPS13C	c.10438C > T	p.Arg3480*	NM_020821.2	Inherited from mother	Nonsense	Heterozygous	chr15	True	8.7	1.0000	1.2411	NA
P124	Proband and parents	FIBP	c.86-2A > C	NA	NM_198,897.1	Inherited from mother	Splice acceptor	Heterozygous	chr11	True	40.7	0.0077	Not estimable	Linked
		KIF20B	c.4180A [2 > 1]	p.Asn1394Thrfs*30	NM_016195.2	Inherited from mother	Frameshift	Heterozygous	chr10	True	10.2	1.0000	1.8631	NA
		LRRFIP1	c.1702A [6 > 7]	p.Glu570Argfs*11	NM_004735.3	Inherited from mother	Frameshift	Heterozygous	chr2	True	7.3	0.0074	Not estimable	NA
		LMF1	c.468C [2 > 1]	p.Leu157Serfs*57	NM_022773.2	Inherited from mother	Frameshift	Heterozygous	chr16	True	5.8	0.0497	7.4761	NA
		ZNF700	c.415dupT	p.Ser139Phefs*4	NM_144,566.1	Inherited from mother	Frameshift	Heterozygous	chr19	True	17.3	0.2864	3.7321	NA
P130	Proband and parents	BTBD1	c.655delA	p.Ile219Leufs*3	NM_025238.3	Inherited from mother	Frameshift	Heterozygous	chr15	True	NA	0.3061	0.0000	NA
		NUP37	c.239_245delGGAGCCC	p.Trp80*	NM_024057.2	Inherited from mother	Frameshift	Heterozygous	chr12	True	17.8	0.0286	Not estimable	Linked
		SLC35D2	c.47_48insG	p.Gly17Argfs*69	NM_007001.2	De novo	Frameshift	Heterozygous	chr9	False	5.1	0.1423	7.4761	NA
		ULK4	c.1797G > A	p.Trp599*	NM_017886.2	Inherited from mother	Nonsense	Heterozygous	chr3	True	27.5	0.0018	Not estimable	Not Linked
P131	Proband and parents	ACSF2	c.92G > A	p.Trp31*	NM_025149.4	Inherited from mother	Nonsense	Heterozygous	chr17	True	4.5	0.0308	Not estimable	NA
		ADAM8	c.1948+1G > A	NA	NM_001109.4	De novo	Splice donor	Heterozygous	chr10	False	2.3	0.5901	0.5310	NA
		CWF19L2	c.308A [7 > 8]	p.Asn105Lysfs*3	NM_152,434.2	Inherited from mother	Frameshift	Heterozygous	chr11	True	9	0.2921	3.7321	NA
		DDX39B	c.736-1G > C	NA	NM_004640.6	De novo	Splice acceptor	Heterozygous	chr6	False	21.3	0.0004	7.6434	Linked
		GLIPR1L2	c.97_110delCTACTGCTACTGGG	p.Leu33Phefs*2	NM_001270396.1	Inherited from mother	Frameshift	Heterozygous	chr12	True	44	0.2826	3.7321	NA
		KIAA1549L	c.33_45delTCTCATAGGCATC	p.Gly14*	NM_012194.2	Inherited from mother	Frameshift	Heterozygous	chr11	True	1.3	0.2872	3.7321	NA
		PIGT	c.1034-1G > C	NA	NM_015937.5	Inherited from mother	Splice acceptor	Heterozygous	chr20	True	34.5	0.0308	Not estimable	Linked
		PPP1R3C	c.145C > T	p.Arg49*	NM_005398.5	Inherited from mother	Nonsense	Heterozygous	chr10	True	2.2	0.0308	Not estimable	NA

HGVS, Human Genome Variation Society; NA, not available.

These six novel genes potentially accounted for a fraction (3.76%, 5 out of 133 patients) of the heritability of NOA in question. Combined with the three known genes (yellow), the six novel genes potentially accounted for an overall 6.77% (9 out of 133 patients) heritability of NOA in this study. In addition, the 19 following genes (*p* < 0.05; OR > 1 or values not estimable; NE values not less than 1) were also regarded as expansive candidate genes for each proband, including *NDUFAF1* for patient 38, *TMEM63A* and *SEMA3F* for patient 52, *BICC1*, *SPRR2F*, and *GSTM3* for patient 60, *ABCB8*, *PRRG4*, *RPUSD4*, and *ZNF221* for patient 62, *SELP* and *GNPTAB* for patient 98, *DDX11* and *POLD2* for patient 117, *LRRFIP1* and *LMF1* for patient 124, *ULK4* for patient 130, *ACSF2* and *PPP1R3C* for patient 131.

## Discussion

Whole exome-based RVAS analysis identified a series of genes associated with NOA in this study. Especially, the most significant proteins, WARS1 (tryptophanyl-tRNA synthetase), DHRS4 (dehydrogenase/reductase 4), and RRBP1 (ribosome binding protein 1), were also involved in the meiosis related pathway, indicating they are associated with NOA. Meanwhile, three previously reported genes, *TDRD7*, *SYCP3*, and *BRCA2*, were also identified by RVAS and involved in the meiosis-related pathway, which partially supported the efficacy of the RVAS test flow in this study.

Combining RVAS analysis and trios linkage analysis showed the overlapped genes, *ACTL8* (encodes actin-like 8 protein, restricted expression toward testis), *TRA2B* (encodes transformer 2 beta homolog protein), *IDE* (encodes insulin-degrading enzyme), *FIBP* (encodes FGF1 intracellular binding protein), *NUP37* (encodes nucleoporin 37, a constituent of the nuclear pore complexes required for mitosis and probably for meiosis) (Loiodice, Alves et al., 2004), and *PIGT* (encodes phosphatidylinositol glycan anchor biosynthesis class T protein), were associated with NOA, as well as involved in the meiosis-related PPI network. Especially, the gene *NUP37* was previously identified as indispensable for mitosis, we speculate that *NUP37* (p.Trp80*, a frameshift mutation found in one out of 133 patients while absent in controls) may also play important roles during meiosis and spermatogenesis. In addition, the expression of *ACTL8* gene is restricted in testis, the actin-like 8 protein ACTL8 (p.Val254Aspfs*4, a frameshift mutation found in one out of 133 patients, while being absent in controls) is assumed to execute the functions during spermatogenesis and play a role in NOA.

The other genes identified by RVAS or parental-proband trios analysis, especially those involved in the meiosis-related PPI network like *PIWIL2*, *HNRNPC*, and *DDX39B* may also play a role in NOA. It has not escaped our notice that *PIWIL1* (encodes Human P-element-induced wimpy testis 1 protein, a paralog of human PIWIL2) was a previously identified gene causative for NOA; Human P-element-induced wimpy testis (PIWI) proteins act as protectors of germline, and are expressed mainly in the germline cells (Gou, Kang et al., 2017), the phenotype of *Piwil2*-deficient (knockout) male mice exhibited azoospermia with complete spermatogenic arrest according to the Mouse Genome Database, hence *PIWIL2* (p.Gln11Serfs*78, a frameshift mutation found in one, P129, out of 133 patients while absent in controls) may play a similar role during spermatogenesis and in NOA.

Despite significant advances have been achieved in understanding the etiology of NOA, this study has certain limitations. First, prospectively more functional evidence like the cellular or physiological experiments should be investigated to address this question better. Second, the limitation lies in that non-LOFs like the missense mutations and small insertions or deletions which may be pathogenic were not considered. Finally, the false-positive rate of the *de novo* mutations (Table 2) is relatively high, which is in part attributable to sequencing errors; therefore, we suggest that *de novo* mutations should be treated with caution in clinical practice.

We demonstrate that RVAS identified a pool of genes, especially the most significant novel genes *WARS*, *DHRS4*, and *RRBP1* which were also involved in the meiosis-related PPI network, indicating they are associated with NOA. In addition, six novel genes, *ACTL8*, *TRA2B*, *IDE*, *FIBP*, *NUP37*, and *PIGT* were revealed to be associated with NOA in both the RVAS and trios linkage analysis, as well as involved in the meiosis-related pathway; we conclude that these six novel genetic risk factors potentially account for a fraction (3.76%, 5 out of 133 patients) of the heritability of NOA in question, together with the three previously reported genes would potentially account for 6.77% (9 out of 133 patients) heritability of NOA in question in this study.

This study not only sheds light on the underlying pathological mechanism of NOA, but also offers valuable insight into the genomic landscape of NOA to constitute a potential basis for more efficient diagnosis yield in the future clinical application, and might have broad implications on men’s health.

## Materials and Methods

### Study Design and Patients

From this center, 133 patients with idiopathic NOA and large sets of controls (343 external male and 22 family-based controls) were recruited. Of 133 patients, 11 cases together with their parents were enrolled as trios. All patients underwent semen analysis at least on three different occasions, whereas no sperm were observed in the ejaculate even after centrifugation, and those with a history of orchitis, obstruction of vas deferens, or endocrine disorders were excluded. All the controls had fathered at least one child.

The clinical characteristics of the patients were summarized in Supplementary Table S1. These patients were selected according to the following criteria: 1) azoospermia due to either spermatogenic arrest (at spermatogonial or spermatocyte level) or SCOS (Supplementary Figure S1); 2) normal karyotype; 3) absence of Y-chromosome microdeletions and *TEX11* mutations (including the copy number variation (CNV) of *TEX11*); and 4) absence of a list (Supplementary Table S2) of known causes for azoospermia.

### Genomic Analysis

The genomic DNA was extracted from the peripheral blood. All the WES data underwent the same quality control filtering and pruning procedures to maximize parity between cases and controls. The average sequencing depth of whole-exome target regions for each sample was higher than 100, and ×20 coverage for more than 95% targeted bases was achieved for each sample. The process of bioinformatics analysis includes data filtering, alignment, mutation detection, and result annotation. The raw data were first evaluated for quality to remove low-quality and adapter contaminated reads. The clean data containing pair-end reads were mapped to the human genome (NCBI37/hg19 genome assembly) using BWA software (Burrows-Wheeler Aligner http://sourceforge.net/projects/bio-bwa/, version 0.7.15). The PCR-induced duplication was eliminated using Picard software. SNVs and Indels were tested using the genomic analysis toolkit (GATK). The GATK pipeline was used to identify the variations. For subsequent analyses, variants with minor allele frequencies (MAF) lower than one percent or uncatalogued in the Genome Aggregation Database (gnomAD) and the 1,000 Genomes were annotated with ANNOVAR. Loss of function (LOF) variations were defined as frameshift mutations, initiation mutations, premature or stopless mutations, or disruption of canonical splicing sites (± 2 bp). CNVs were analyzed using ExomeDepth and CNVkit. PPI network was built by the search tool for the retrieval of interacting genes/proteins (STRING) and further analyzed by Cytoscape_v4.0.2 software.

For linkage analysis, according to a maternally inherited pattern or the *de novo* mutation mechanism to which the gender-specific disease NOA condition was supposed to be genetically attributed, the paternally inherited variations were excluded; meanwhile, the probands’ LOFs were preserved only if the LOFs were not found in the 343 male controls. Sanger sequencing was performed for validating the variations.

### Statistical Analysis

A total of 282 LOFs were removed due to the exact test for Hardy-Weinberg equilibrium (*p* < 0.05). The remaining LOFs were analyzed in the gene burden association test using the sequence kernel association test (SKAT) package (Ionita-Laza, Lee et al., 2013). The two-tailed *p* values were calculated, *p* < 0.05 was considered as statistically significant. False discovery rate (FDR) was calculated for PPI network enrichment analysis. The resulting *p* values were adjusted using Benjamini and Hochberg’s approach for controlling the false discovery rate. The odds ratio (OR) and 95% confidence interval (CI) were calculated based on the genotype matrix. The quartile–quartile plot (Q–Q plot) for the RVAS analysis was drawn (Supplementary Figure S2). All analyses were performed using R v4.0.2.

## Data Availability

The datasets presented in this study can be found in online repositories. The name of the repository and accession number can be found below: CNSA (https://db.cngb.org/cnsa/); CNP0001080. The Human Protein Atlas database, http://www.proteinatlas.org/ 1,000 Genomes, http://www.internationalgenome.org/ Genome Aggregation Database, https://gnomad.broadinstitute.org/ Mouse Genome Database, http://www.informatics.jax.org/.

## Supplementary Material

Supplementary Figure S1 The histopathology examination for patients with NOA (spermatogenic arrest or Sertoli cell-only syndrome). The hematoxylin-eosin (HE) staining was performed to facilitate the diagnosis for patients. The two upper images showed a patient diagnosed as Sertoli cell-only syndrome; and the two lower photos showed another patient diagnosed as spermatogenic arrest type I. Only Sertoli cells, or primary spermatocytes, or spermatogonium could be detected, whereas no mature spermatozoa could be seen in either case. The red arrow indicated the corresponding cell types.

Supplementary Figure S2 The quartile–quartile plot (Q–Q plot) for the RVAS analysis. 95% CI was shown as the blue solid line around the uniform distribution line (red dashed line).

Supplementary Figure S3 The histopathology examination for 5 independent patients with NOA. The hematoxylin–eosin (HE) staining was performed to facilitate the diagnosis for patients P62, P117, P124, P130, and P131, respectively. The red arrow indicated the corresponding cell types.
