# Supplementary figures and images for: Whole Exome Sequencing Identifies Genes Associated With Non-Obstructive Azoospermia
Source: Front Genet. 2022 Apr 13;13:872179. doi: 10.3389/fgene.2022.872179 (PMC9043847; doi:10.3389/fgene.2022.872179)

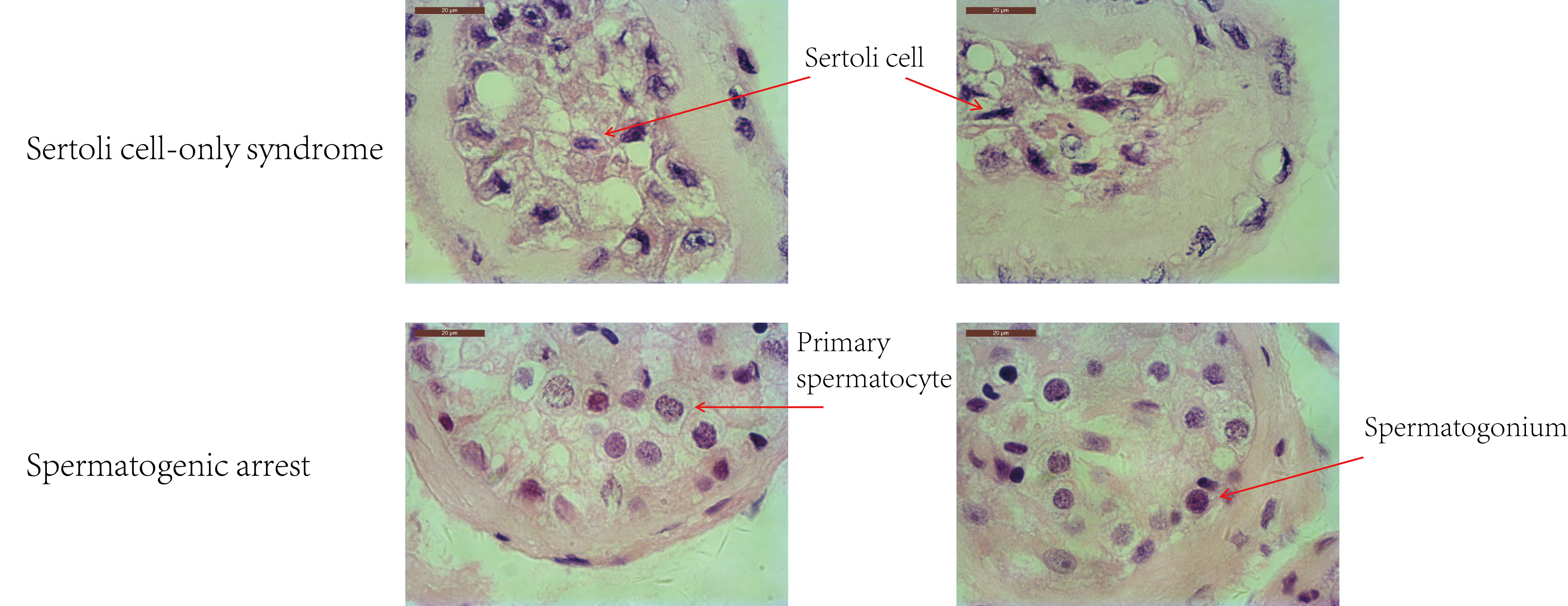

Supplement: Supplementary file 3 [file Image1.JPEG]

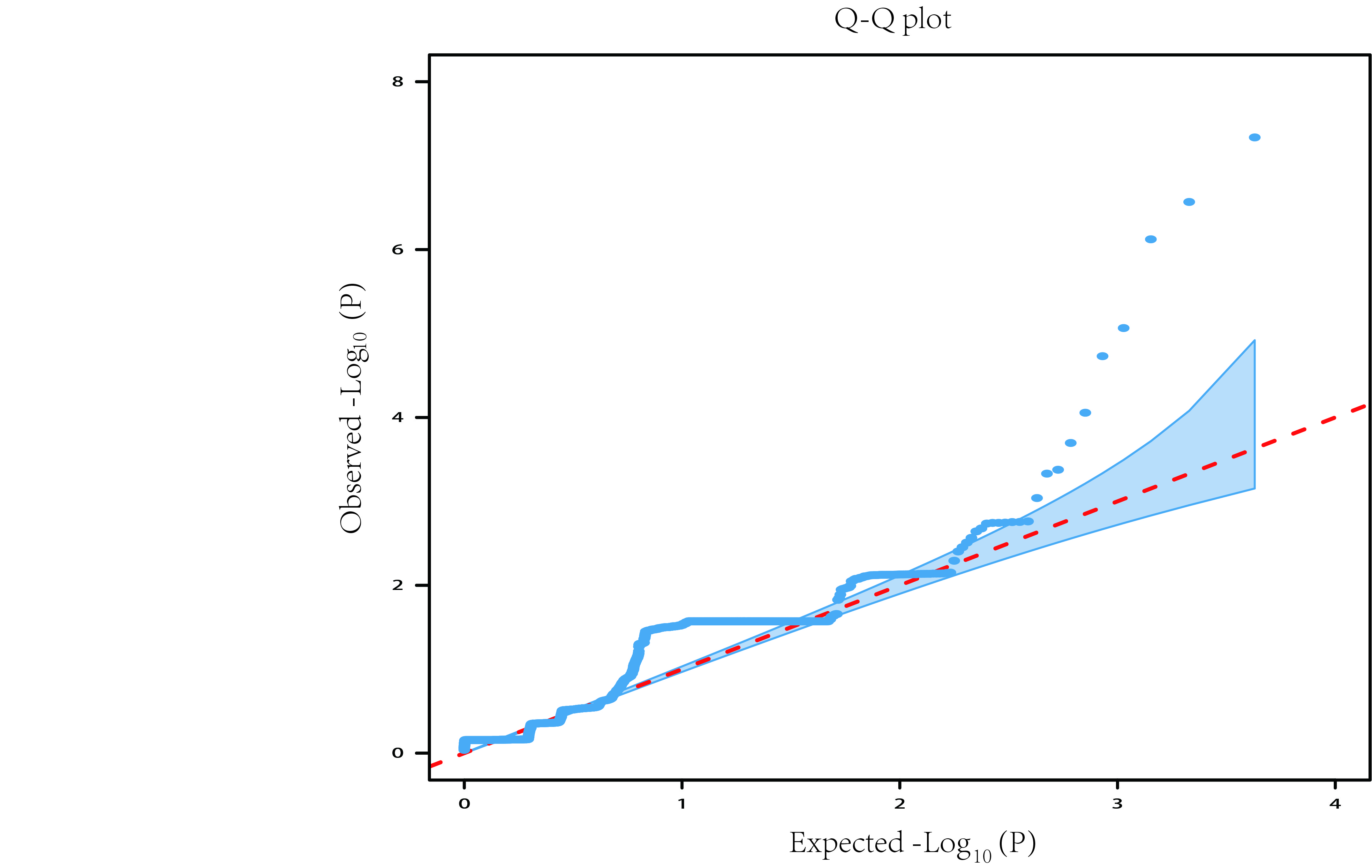

Supplement: Supplementary file 4 [file Image2.JPEG]
