# Supplementary material for: Whole Exome Sequencing Identifies Genes Associated With Non-Obstructive Azoospermia
Source: Front Genet. 2022 Apr 13;13:872179. doi: 10.3389/fgene.2022.872179 (PMC9043847; doi:10.3389/fgene.2022.872179)

P62

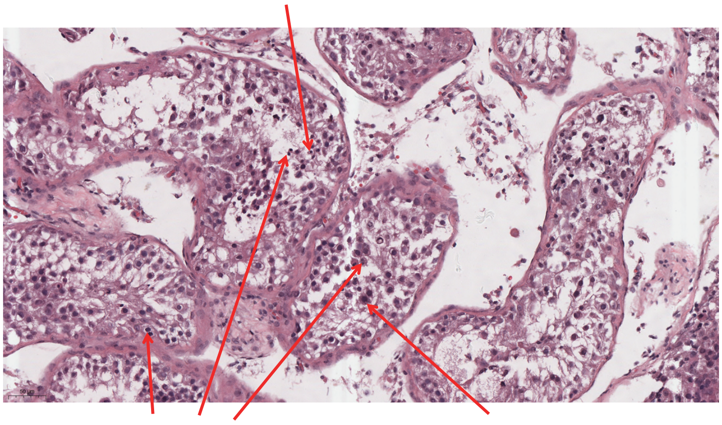

P124

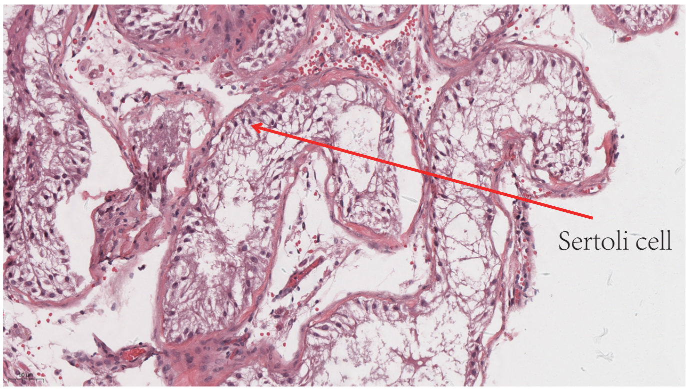

P117

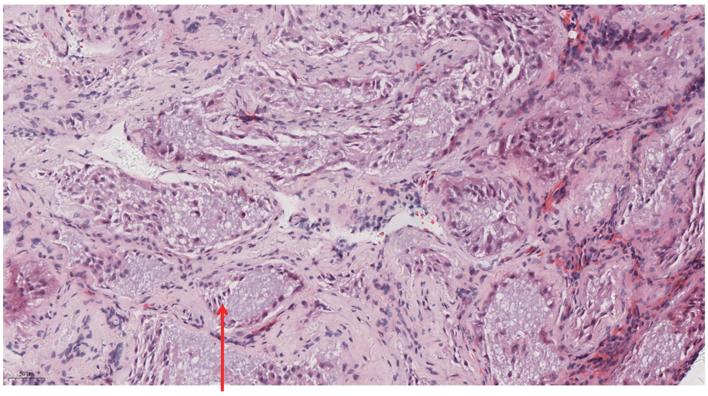

P130

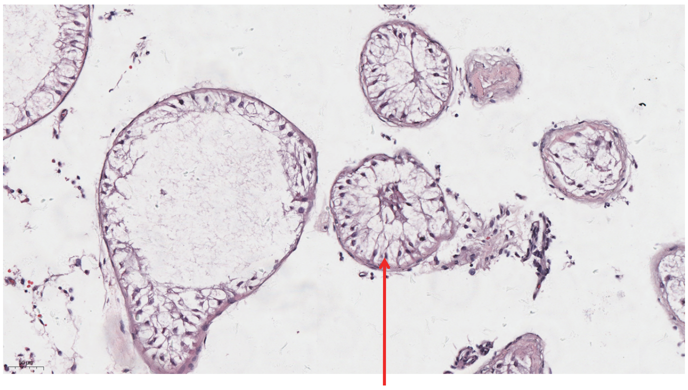

P131

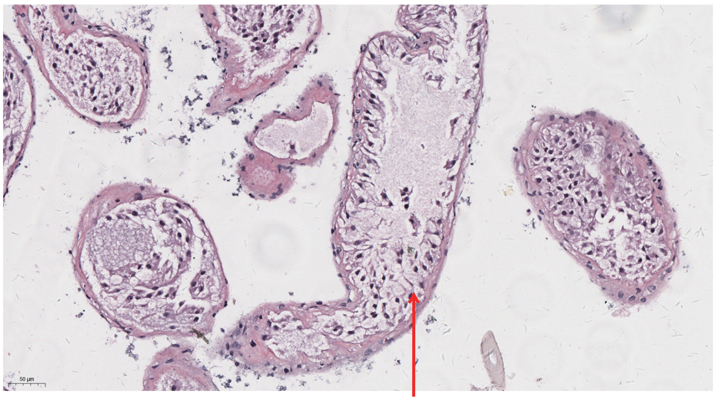

Supplement: Supplementary file 5 [file Image3.PDF]
